# Construction and Characterization of Immortalized Fibroblast Cell Line from Bactrian Camel

**DOI:** 10.3390/life13061337

**Published:** 2023-06-07

**Authors:** Meilin Yan, Fang Yong, Wangye Ji, Lili Zhang, Shuqin Zhao, Yuan Gao

**Affiliations:** 1 College of Life Science and Technology, Gansu Agricultural University, Lanzhou 730070, China; 2 Gansu Key Laboratory of Animal Generational Physiology and Reproductive Regulation, Lanzhou 730070, China

**Keywords:** Bactrian camel, fibroblast, BCF23, hTERT, VIM

## Abstract

Immortalized cell lines with many advantages are widely used in various experimental contexts by many different labs. However, the absence of available cell lines poses difficulties for research in some species, such as camels. To establish an immortalized Bactrian camel fibroblast (iBCF) cell line and understand its biological characteristics, primary fibroblast cells from Bactrian camels were isolated and purified using enzymatic digestion in this study, and telomerase reverse transcriptase (hTERT) vectors were introduced into primary BCF (pBCF) for continuous passage to 80 generations after screening with G418. The cell morphology of different generations was examined under a microscope. Cell cycle and viability were evaluated by flow cytometry and CCK-8 assay, respectively. Cellular genes expression was monitored by qPCR, immunofluorescence, and Western blot, respectively. Chromosomes were determined by karyotyping. The results showed that like most other cells, both pBCF and iBCF were sensitive to nutrient concentrations and adapted to culture in the medium with 4.5 g/L glucose and 10% fetal bovine serum (FBS) concentration. hTERT gene was introduced and stably expressed in iBCF cells, which promoted BCF cell immortalization. The fibroblast specific marker vimentin (VIM) is expressed in both pBCF and iBCF, but epithelial marker cytokeratin18 (CK18) expression is weak in BCF cells. Proliferation and viability detection showed that hTERT-induced iBCF exhibits faster growth rates and higher viability than pBCF. Karyotyping showed that iBCF maintained the same number and morphology of chromosomes as the pBCF. This study demonstrated that we have successfully constructed an immortalized Bactrian camel fibroblast cell line, which was named BCF23. The establishment of the BCF23 cell line provides a foundation for expanding camel-related research.

## 1. Introduction

Camels, with the ability to survive under drought and chronic hunger, developed exceptional anatomical, physiological, and biochemical metabolic characteristics [1]. The limited lifespan of cells in in vitro culture is a significant factor that can impact the stability of research findings, particularly for animals with limited sample and cell resources like camels [2]. The establishment of immortalized cell lines is an effective way to solve these problems [3]. Fibroblast is a common type of cell with strong growth and division capabilities, and is widely available for experimental research. However, they also face problems such as cell senescence and the inability to pass multiple generations due to telomere shortening [4]. Telomeres, located at the ends of chromosomes and composed of multiple DNA repeat sequences of varying lengths and protein complexes [5], play a crucial role in maintaining chromosome stability, preventing chromosome enzyme degradation, and promoting normal cell growth [6]. Telomeres deficiency can cause chromosome degradation, recombination, and changes in chromosome coding genes, resulting in sticky chromosomes, changes in structure and function, and ultimately abnormal chromosome replication and cell death [7].

Telomerase is an enzyme involved in telomere synthesis and is particularly important in synthesizing telomere repeat sequences and maintaining telomere length [8]. Normal somatic cells have limited passaging due to telomere shortening, which indicates the crucial protective role of telomeres for chromosomes [9]. Immortalized cell lines can be established through several methods, such as spontaneous immortalization [10], mutation of p53 and pRb genes, overexpression of viral oncogenes [11,12], radiation [13], viral transformation, e.g., using SV40 virus or EBV, and ectopic expression of telomerase reverse transcriptase (TERT) [14,15]. Among these methods, the most commonly used and extensively studied one is the overexpression of human TERT (hTERT) [16,17].

Previous studies have shown that the hTERT gene can be introduced into tissue cells of different species, including mesenchymal stem cells in dairy goats and embryonic fibroblasts in mice [18,19,20,21], resulting in the establishment of an immortalized cell line. These cell lines remain stable in terms of their biological characteristics even after undergoing 50–80 passages [3]. However, there is limited knowledge regarding the establishment of immortalized camel cell lines. The objective of this study is to utilize the hTERT plasmid induction method to construct immortalized camel fibroblast cell lines, termed BCF23. The successful construction of a camel fibroblast cell line is indicated by cell morphology, marker proteins, and proliferation activity tests. This cell line will provide stable cell materials for research related to camels.

## 2. Materials and Methods

### 2.1. Cells and Reagents

Fresh Bactrian camel tissue for isolation of primary fibroblasts was collected from a slaughterhouse in Zhangye (China). Bovine mammary epithelial cells MAC-T were preserved in our laboratory. The pCI-neo-hTERT plasmids were obtained by our laboratory. DMEM medium, phosphate-buffered saline (PBS, pH = 7.2), trypsin-EDTA solution (0.25%), penicillin, and streptomycin (100×) were purchased from BasalMedia (Shanghai, China). Collagenase IV, RIPA protein lysis buffer, TRIzol, CCK-8, propidium iodide (PI), and a BCA protein detection kit were purchased from Beyotime (Beijing, China). An ECL chemiluminescence detection kit was purchased from NeoBioscience (Suzhou, China). A reverse transcription kit, the SYBR Premix Ex Taq II Kit, G418, and protein markers were purchased from TaKaRa (Dalian, China). Fetal bovine serum (FBS) was purchased from Gibco (Carlsbad, CA, USA). Lipofectamine 2000 transfection reagent, a plasmid extraction kit, restriction enzymes, rabbit anti-hTERT polyclonal antibody, Alexa-Fluor 488 labeled goat anti-Rabbit IgG, and HRP-conjugated goat anti-rabbit IgG were all purchased from ThermoFisher Scientific (Waltham, MA, USA). Rabbit anti-β-actin antibody, anti-VIM polyclonal antibody, and anti-CK18 polyclonal antibody were obtained from Proteintech (Chicago, IL, USA). PCR primers were synthesized by Sangon Biotech (Shanghai, China).

### 2.2. Isolation and Culture of Primary Camel Cells

Epididymis tissue of 2-year-old healthy Bactrian camel was collected for isolation and culture of primary cells in a sterile environment. Briefly, epididymal epithelium was cut into small pieces and washed with PBS and complete medium containing 10% FBS and dual-antibiotics, respectively, until the suspension was clear. Then, twice the volume of collagenase IV was added, and the mixture was placed in 37 °C for 1.5 h. The digestion was terminated by adding complete medium, and the mixture was then centrifuged at 1000 rpm for 5 min. After discarding supernatant, the precipitate was resuspended using a suitable amount of complete culture medium, and the mixture was seeded into a new culture flask and placed in a 37 °C, 5% CO_2_ humidity incubator for growth. The culture medium was replaced every 2 d, and the cell state was observed under an inverted microscope (Olympus, Hamburg, Germany).

### 2.3. Cell Culture and Passage

Cells were maintained with DMEM medium containing 10% FBS and dual-antibiotics in a 37 °C, 5% CO_2_ condition. The culture medium was replaced every 2–3 d during the cell culture process. After cells reached the confluence, the cells were passaged using 0.25% trypsin/EDTA.

### 2.4. Cell Transfection and G418 Screening

Primary camel fibroblasts incubated in 60 mm dishes with 70~80% confluence at passage 3 were transfected with pCI-neo-hTERT plasmid using Lipofectamine 2000 reagent following the manufacturer’s protocol. For G418 screening, the camel fibroblasts after transfection were seeded in 96-well plates at a density of 1000 cells/well. Selection media containing different concentrations of G418 were added to each well for incubating for 2 weeks. The selection media was changed every 2 d. The minimum lethal concentration of G418 for camel fibroblasts was determined to be 400 ng/μL. For positive clones screening, the concentration should be increased by 50 ng/μL. Thus, 450 ng/μL was considered as the optimal concentration for G418 selection of positive clones for camel fibroblast. After all cells in the control group were dead, the concentration of G418 in the culture medium was reduced by half and the screening was continued. After 2 weeks, the growth medium was replaced with G418-free complete medium for cultivation.

### 2.5. Cell Viability and Proliferation Detection

The cell viability and proliferation were evaluated using CCK-8 assay and a cell counting method, respectively. For the CCK-8 assay, cells were seeded in 96-well plates at a density of 1 × 10^3^ cells per well. After treatment with different serum concentrations (0%, 5%, 10%, and 15%) or glucose concentration (0, 2, 4.5, 9 g/L) for different times at 37 °C, CCK-8 of 10 μL solution was added to each well, and the absorbance at 450 nm was measured using a ReadMax 1900 microplate reader (Dukee, Shanghai, China). The data were collected for cell viability analysis. For the cell counting, monolayer cells were taken off using trypsin/EDTA and seeded onto 24-well plates with a density of 1 × 10^4^ cells/well. The cell count was recorded every 24 h using a blood cell counting plate, and continuously for 7 d. The data were collected for plotting growth curves. All experiments were repeated at least three times.

### 2.6. Immunofluorescence Assay

Cells were inoculated in 6-well plates with cover slips to a monolayer [22]. Then cover slips with cells were collected for washing twice with pre-cooled PBS and fixation with 4% paraformaldehyde for 30 min at room temperature. After washing 3 times with PBS, cells were permeabilized in 0.2% Triton X-100 for 10 min and blocked with 5% BSA for 30 min at room temperature, respectively. Cover slips were incubated with rabbit anti-Vimentin polyclonal antibody (1:450) overnight at 4 °C, followed by incubation with an Alexa-Fluor 488 labeled goat anti-Rabbit IgG secondary antibody (1:400) for 1 h at room temperature in the dark. After washing 3 times with PBS, the cells were stained with 4′,6-diamidino-2-phenylindole (DAPI) for 5 min. Images were captured and recorded using an inverted fluorescent microscope (Echo-Labs, Englewood, CO, USA).

### 2.7. Real-Time Quantitative PCR (qPCR) Assay

Total RNA was extracted from camel fibroblasts using the TRIzol method [23]. cDNA was synthesized according to the instructions of the PrimeScript RT Reagent Kit, and the concentration and purity of cDNA were detected using an ultraviolet spectrophotometer. The sequences of the specific primers used are listed as follow. *GAPDH* forward primer: 5′-CTGGTGCTGAGTACGTTGTGGAG-3′ and reverse primer: 5′-AGGAGGCGTTGCTGACAATCTTG-3′, yielding a product size of 186 bp. hTERT forward primer: 5′-CCGATTGTGAACATGGACTACG-3′ and reverse primer: 5′-CACGCTGAACAGTGCCTTC-3′, yielding a product size of 99 bp. The qPCR reaction system was prepared according to the protocol of the SYBR Premix Ex Taq II Kit, and the reaction was performed on the LightCycler instrument (Roche, Indianapolis, IN, USA). Relative gene expression quantity was analyzed using the 2^−ΔΔCt^ method and the amount of transcript in each sample was normalized using *GAPDH* as the internal control.

### 2.8. Western Blot Assay

Cellular total protein of the cells was extracted using RIPA protein lysis buffer and protein concentration was measured using the BCA protein assay kit. Equal total protein was subjected to 12% SDS-PAGE gel for electrophoresis separation, and then electro-transferred onto a 0.45 μm polyvinylidene difluoride (PVDF) membrane (Biosharp, Beijing, China). The membrane was blocked for 2 h with 5% skim milk, followed by overnight incubation at 4 °C with primary antibodies, including anti-β-actin antibody (1:5000), anti-VIM antibody (1:2000), anti-CK18 antibody (1:2000), and anti-hTERT polyclonal antibody (1:1000). After washing with PBS for 3 times, the membrane was co-incubated with the HRP-conjugated goat anti-rabbit IgG (1:8000) for 1 h at room temperature. Finally, protein bands were visualized using the ECL chemiluminescence detection method and captured by the Gel imaging system (Tannon, Shanghai, China).

### 2.9. Cell Cycle Detection

Cells were harvested by digestion with trypsin without EDTA when the cell convergence reached 70%~80%, and fixed with pre-cooled 75% ethanol overnight at 4 °C. Then, PI staining was performed according to the previous description [24]. Briefly, cells were incubated with PI staining solution at 37 °C in the dark for 30 min. Then cells were resuspended and filtered through a 40 μm cell strainer. The intensity of the fluorescent signal was acquired using a FACSAria (BD Biosciences, San Jose, CA, USA). Flow cytometry data was analyzed using Modfit LT software (version 5.0, Bedford, MA, USA).

### 2.10. Karyotype Analysis

Karyotyping was performed to determine the chromosome number. Briefly, both pBCF and iBCF cells of 5 × 10^4^ were inoculated in a T25 flask with 5 mL culture medium. After reaching 90% confluence, cells were incubated with Colchicine (0.3 μg/mL, Gibco, Waltham, MA, USA) at a 37 °C for 3 h. Cells were taken off using trypsin/EDTA, followed by the addition of complete media and centrifugation at 1300 rpm for 10 min. Then the cells were suspended in 0.075 mol/L KCl hypotonic solution and maintained at 37 °C for 20 min. Cells were fixed using 8 mL fresh ice-cold acetic acid/methanol (1:3, *v*/*v*) solution three times. After centrifugation at 1300 rpm for 10 min, the cells were resuspended using 0.5 mL fixation solution and dropped onto ice-cold glass slides at a height of 0.3 m. Slides were dried at 70 °C for 3 h and stained in Giemsa solution for 5–10 min. Chromosomes were observed and photographed under the 1000× magnification under the bright-field microscope (Nikon, Tokyo, Japan). The diploid number of chromosomes was counted for passage 5 (P5) metaphases of pBCF and P75 metaphases of iBCF. The routine staining karyotyping was performed for P5 metaphases of pBCF and P75 metaphases of iBCF.

### 2.11. Statistical Analysis

The data were presented as mean ± standard deviation (SD) and analyzed using SPSS 19.0 statistical software. The significance of the differences in each group was analyzed with a two-tailed Student’s *t*-test. *p*-values of < 0.05 were considered to be significant.

## 3. Results

### 3.1. Morphological Characteristics of Primary Bactrian Camel Fibroblast (BCF)

**Figure 1:**
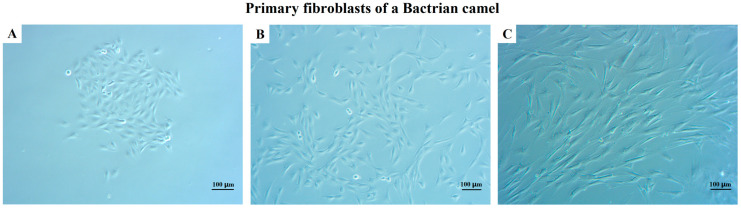
Cell morphology of primary BCF (pBCF). (**A**) Morphology of newly adherent pBCF. (**B**) Morphology of third-generation pBCF. (**C**) Morphology of 12th generation pBCF. Scale bars: 100 μm.

To obtain primary BCF (pBCF) for in vitro culture, fresh camel epididymis tissue was collected for primary cell separation using a collagenase digestion method. The cell morphology was observed under an inverted microscope. As shown in Figure 1, dispersed round cells digested by collagenase quickly adhered to the bottom of flask after 4 h and reached complete adhesion after 24 h cultivation. The cell morphology was mainly spindle-shaped, although the cell size varies. There was a small number of cells of other shapes, such as triangles, irregular shapes, etc., mixed in the spindle cell population (Figure 1A), which may be other types of cells, such as epithelioid cells.

Compared to epithelioid cells, fibroblasts are more sensitive to trypsinization. A gentle trypsinization will remove fibroblasts but leave epithelioid cells behind [25]. To obtain pure fibroblasts, this study used a method of controlling trypsinization time to harvest fibroblasts and remove epithelioid cells during passaging. As shown in Figure 1B, after 2–3 passages, the cells all grew into spindle-shaped cells with uniform morphology and size, and the cells exhibited a strong sense of three dimensionality, indicating that this study obtained pure primary fibroblasts. However, after 10 generations, the cells showed signs of aging, such as abnormal elongation, cytoplasmic granules, cell deformation, and decreased vitality, and eventually stopped dividing (Figure 1C). The result indicated that primary BCF was isolated and purified successfully. The pBCF of passages 2–5 were cryopreserved in the freezing media containing 10% dimethyl sulfoxide (DMSO) and 50% FBS in DMEM at −196 °C in liquid nitrogen.

### 3.2. Construction of Immortalized BCF Cell Line

**Figure 2:**
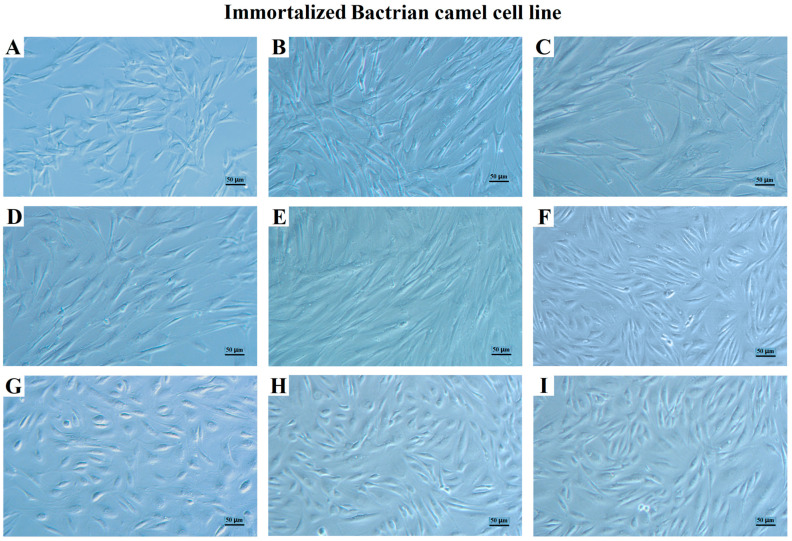
Morphology of iBCF cells in different generations after introducing exogenous hTERT gene. Morphology of 5th generation P5 (**A**), P10 (**B**), P20 (**C**), P30 (**D**), P40 (**E**), P50 (**F**), P60 (**J**), P70 (**H**), P80 (**I**), respectively. Scale bars: 50 μm.

To obtain the immortalized BCF (iBCF) cell line, this study adopted the method of artificially introducing an exogenous hTERT gene to promote cell immortalization. Plasmids carrying the hTERT gene were introduced into pBCF. After screening via G418 with optimal concentration of 450 ng/μL, the BCF cells achieved continuous passage to over 80 generations. As shown in Figure 2, after introducing the exogenous hTERT gene, cells could overcome division limits and continue to divide. The iBCF exhibited a typical long spindle shape with uniform cell morphology throughout each generation, suggesting that the introduction of the exogenous hTERT gene immortalized primary cells; it was tentatively hypothesized that iBCF was successfully constructed. The iBCF of every 10 generations (P10, P20, P30, …) were cryopreserved in the freezing media containing 10% DMS) and 50% FBS in DMEM at −196 °C in liquid nitrogen.

### 3.3. Determination of BCF Culture Conditions

**Figure 3:**
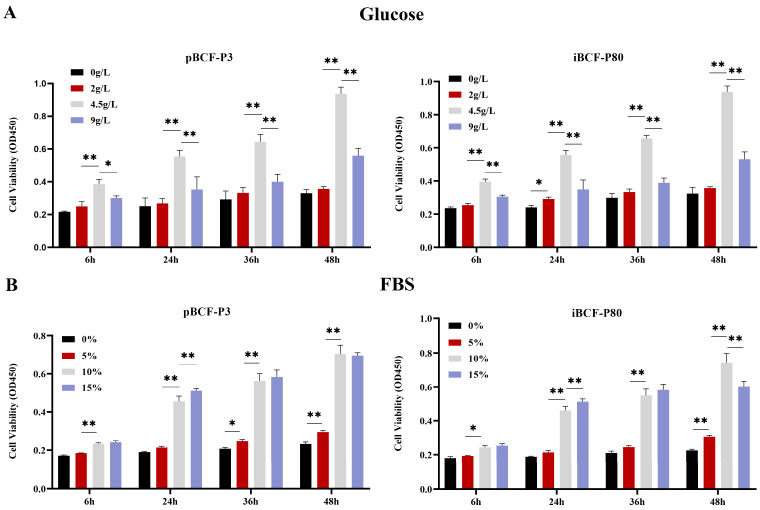
Nutritional conditions for BCF cell growth. (**A**) pBCF and iBCF cells were maintained in culture medium with different glucose concentrations (0, 2, 4.5, 9 g/L) for different times. (**B**). pBCF and iBCF cells were maintained in culture medium with different FBS concentrations (0%, 5%, 10%, and 15%) for different times. pBCF: primary Bactrian camel fibroblast. iBCF: immortalized Bactrian camel fibroblast. FBS: fetal bovine serum. * *p* < 0.05, ** *p* < 0.01.

Due to the scarcity of camel cell resources, we set to determine the culture conditions of BCF and to understand the growth effects of BCF under different nutritional conditions. pBCF and hTERT-induced iBCF cells were cultured to maintain in the medium with different FBS concentrations (0%, 5%, 10%, and 15%) and glucose concentrations (0, 2, 4.5, 9 g/L) for different times, respectively. Cell viability was monitored by CCK-8 assay. As shown in Figure 3, both pBCF (P3) and iBCF (P80) showed similar growth activity pattern responding to nutrient concentration, including glucose and serum. Both BCFs have the highest growth activity at a glucose concentration of 4.5 g/L, and excessive glucose concentration actually inhibits cell growth (Figure 3A). Similarly, both BCFs achieve the best growth activity when the serum concentration reaches 10% (Figure 3B). The result indicated that BCF, like fibroblasts of other species, is sensitive to nutrient concentrations and has the optimal nutritional conditions of 4.5 g/L glucose and 10% FBS in MEM.

### 3.4. hTERT Gene Is Stably Expressed in Immortalized BCF

**Figure 4:**
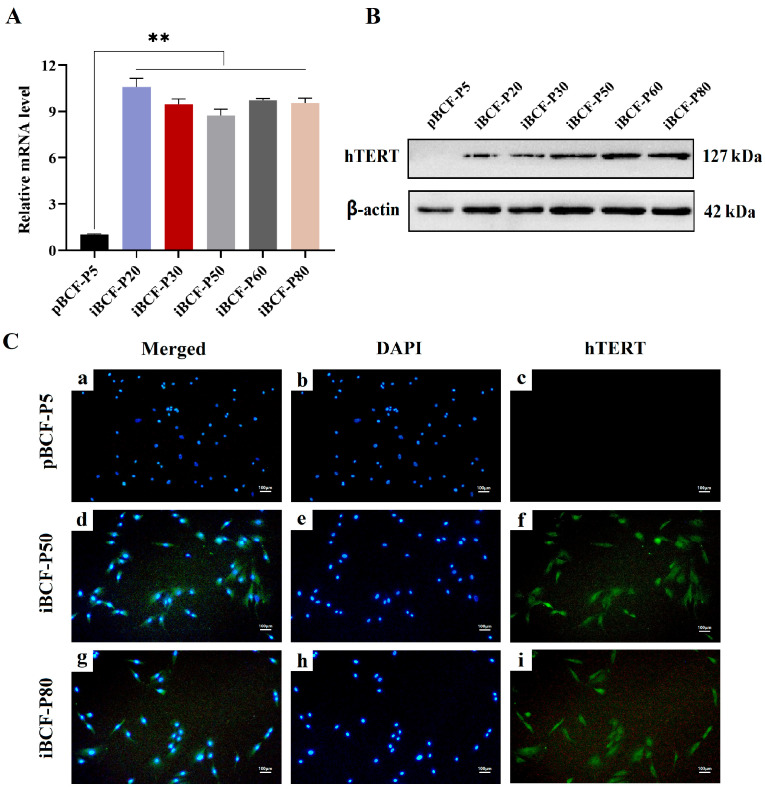
hTERT gene is stably expressed in immortalized BCF. (**A**) qPCR assay showed that hTERT gene was highly expressed in all iBCF cells of different generations at mRNA level. (**B**) Western blot showed that hTERT was expressed in all iBCF cells of different generations at protein level. β-actin was used as a positive control. (**C**) Immunofluorescence signal shown (from right to left): hTERT staining, DAPI staining, and merged (hTERT + DAPI) staining. The top row (**a**–**c**) shows primary BCF passage 5 cells. The middle row (**d**–**f**) and bottom row (**g**–**i**) show immortalized BCF P50 and P80 cells. hTERT protein was stained as green with Alexa-Fluor 488 labeled secondary antibody and nucleus was dyed by blue with DAPI. pBCF: primary Bactrian camel fibroblast. iBCF: immortalized Bactrian camel fibroblast. Scale bars: 100 μm. ** *p* < 0.01.

The introduction of the exogenous hTERT gene promoted BCF cell immortalization. To confirm whether hTERT genes are integrated into the genome for stable expression in the iBCF cell line, the expression and localization of the hTERT gene in iBCF cells was evaluated using qPCR, western blot, and immunofluorescence, respectively. pBCF cells were set as the control. As shown in Figure 4, the hTERT gene expression significantly increased in all iBCF cells of different generations compared to pBCF at the mRNA level (Figure 4A). Consistently, hTERT protein bands appeared in all generations of iBCF cells, but were absent in pBCF (Figure 4B). Additionally, immunofluorescence staining showed that all cells of iBCF P50 and P80 expressed hTERT proteins with green fluorescence, which is mainly distributed in the nucleus, overlapping with DAPI blue; there was also a small amount of hTERT proteins present in the cytoplasm (Figure 4C). In contrast, pBCF cells did not express the hTERT protein. All these results demonstrated that the hTERT gene was introduced and stably expressed in the iBCF cell line, which promoted BCF cell immortalization.

### 3.5. Fibroblast Marker Vimentin (VIM) Is Highly Expressed in Immortalized BCF

**Figure 5:**
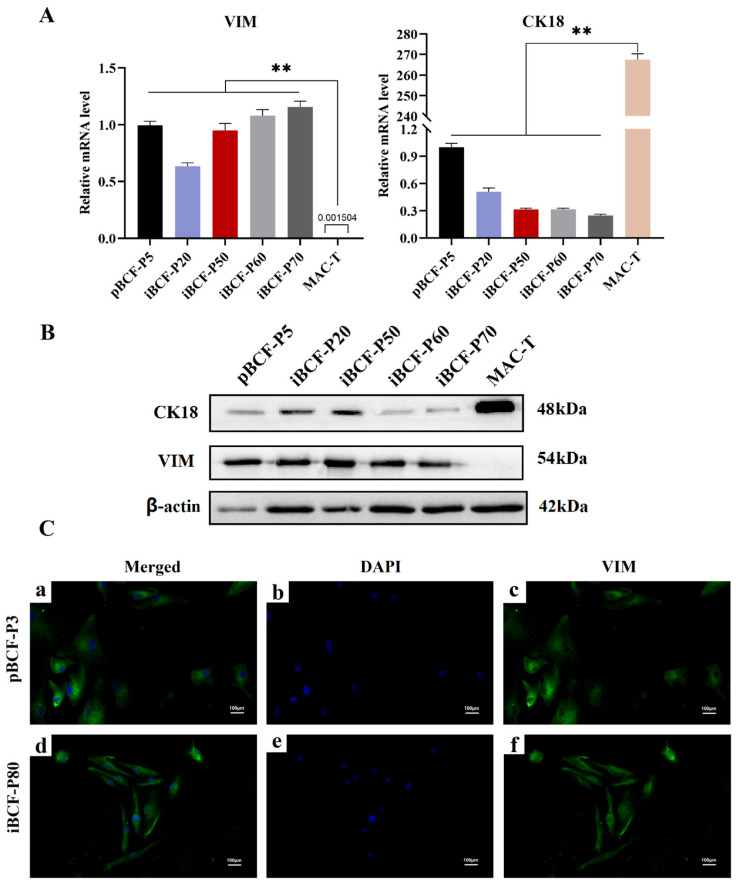
Fibroblast marker VIM is expressed in immortalized BCF cell line. (**A**) qPCR assay showed that VIM is expressed, but CK18 expression is weak in both pBCF and iBCF at mRNA level, compared to MCA-T epithelial cell line. (**B**) Western blot detection showed that VIM is expressed in BCF, but CK18 expression is weak in both pBCF and iBCF at protein level, compared to MCA-T epithelial cell line. β-actin was used as a positive control. (**C**) The images show (from right to left): VIM staining, DAPI staining, and merged (VIM + DAPI) staining. The top row (**a**–**c**) shows primary BCF-P3 cells, and the bottom row (**d**–**f**) shows immortalized BCF-P80 cells. VIM: Vimentin. CK18: cytokeratin 18. MAC-T: bovine mammary epithelial cell. pBCF: primary Bactrian camel fibroblast. iBCF: immortalized Bactrian camel fibroblast. Scale bars: 100 μm. ** *p* < 0.01.

VIM is one of the specific markers of fibroblasts [26], and cytokeratin 18 (CK18) is one of the epithelial cell markers. To further confirm that BCFs are fibroblasts, this study performed VIM and CK18 expression detection using qPCR, western blot, and immunofluorescence stain, respectively. As shown in Figure 5, VIM is expressed in all BCFs, including pBCF and iBCF of different generations, compared to epithelioid cells MAC-T, at both the mRNA level and the protein level (Figure 5A,B). However, CK18 expression is very weak in both pBCF and iBCF, compared to epithelioid cells MAC-T (Figure 5A,B). Immunofluorescence staining showed that VIM is expressed in BCF and widely distributed in cytoplasm (Figure 5C). The result demonstrated that this study obtained pure camel fibroblast cell line, iBCF.

### 3.6. The Growth Characteristic of iBCF Cell Line

**Figure 6:**
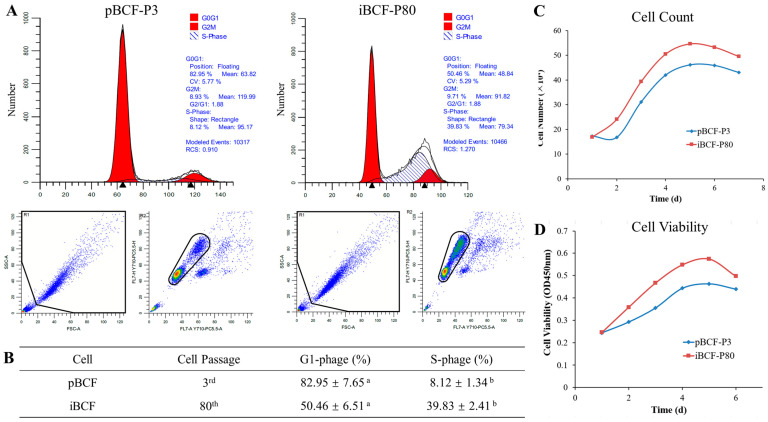
The growth characteristic of immortalized BCF. (**A**) Flow cytometry was performed to evaluate cells number at different stages of the cell cycle of pBCF and iBCF. Red and shadow represent the number of cells in the interphase and division stages, respectively. Blue dots individual cells. The cells in the black box are effective cell populations. (**B**) Cell cycle detection by flow cytometry showed that iBCF had a higher proportion of S phase cells than pBCF, indicated on the graph by shaded areas. The superscript lowercase letters a and b in table indicate a significant difference in different groups (*p* < 0.05). (**C**) Cell count result showed that BCF exhibits an S-shaped growth curve and iBCF enters the growth plateau stage faster than pBCF. (**D**) CCK-8 test indicated that iBCF exhibits higher viability than pBCF. pBCF: primary Bactrian camel fibroblast. iBCF: immortalized Bactrian camel fibroblast.

To evaluate the growth characteristic of BCF cell lines, pBCF and hTERT-induced iBCF cells were collected for cell cycle, proliferation, and viability detection using flow cytometry, cell counting, and CCK8 assays, respectively. As shown in Figure 6, the proportion of pBCF in the G1 phase was significantly higher than in iBCF (82.95 ± 7.65% vs 50.46 ± 6.51%, Figure 6A,B), while the iBCF had a higher proportion of S phase cells than pBCF (39.83 ± 2.41% vs 8.12 ± 1.34%, Figure 6A,B), indicating that the iBCF cell line had stronger cell proliferation capacity, a shorter cell cycle, and faster proliferation rates than pBCF. Moreover, the cell result showed that BCF exhibits an S-shaped growth curve. iBCF also exhibits a faster growth rate and enters the growth plateau stage faster than pBCF (Figure 6C). In addition, cell viability testing also indicated that iBCF exhibits higher viability than pBCF (Figure 6D). All the results demonstrated that hTERT-induced iBCF cell line has excellent cellular viability and proliferative activity.

### 3.7. Karyotype Analysis of iBCF

**Figure 7:**
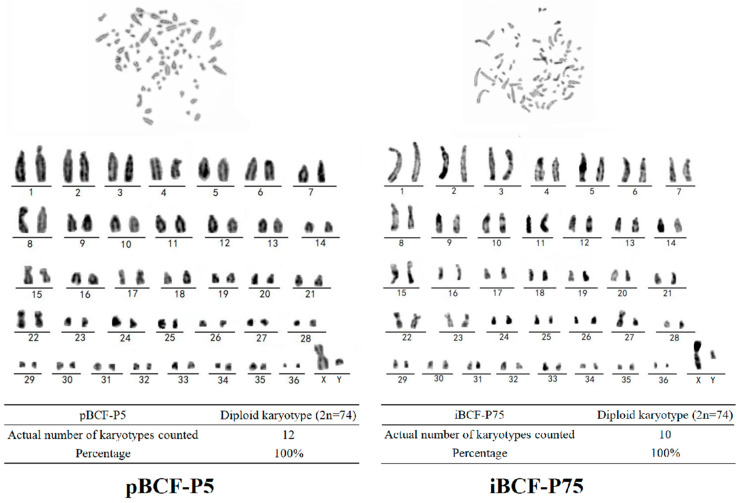
Karyotype analysis showed that iBCF displayed the normal camel chromosome number (2n = 74) and structure as pBCF. pBCF: primary Bactrian camel fibroblast. iBCF: immortalized Bactrian camel fibroblast. 1–36 represent autosomes. X and Y represent sex chromosome.

Karyotyping involves an analysis of the entire chromosome complement through the microscope, which is most useful for detecting cytogenetics changes [27]. To ensure that no chromosomal abnormalities resulted from immortalization of the BCF, karyotype analysis was performed to identify the chromosomal number and structure of iBCF. pBCF was set as the control. As shown in Figure 7, immortal iBCF (P75) showed the normal camel chromosome number (2n = 74) and structure, consistent with a normal chromosome profile of pBCF (Figure 7), indicating that iBCF maintained the normal biological properties.

## 4. Discussion

Camels, with the ability to survive under drought and chronic hunger, developed exceptional anatomical, physiological, and biochemical metabolic characteristics [1]. However, the lack of stable cell lines seriously impacts research on camels. The establishment of immortalized cell lines is an effective way to solve these problems. There are several methods to establish immortalized cell lines, such as spontaneous immortalization, mutation of p53 and pRb genes, overexpression of viral oncogenes [28], radiation, viral transformation, e.g., using SV40 virus or EBV, and ectopic expression of TERT. Among these methods, the most commonly used and extensively studied one is the overexpression of hTERT [16,17]. Actually, the hTERT-induction method has been successfully used to construct multiple cell lines in different species [18,19,20,21,29,30]. In this study, the hTERT-induction method was performed to construct a camel-derived cell line.

This study isolated and purified fibroblasts from camel epididymal tissue. After about 10 passages, however, the primary fibroblasts gradually stopped growing and dividing (Figure 1), which was mainly due to telomere shortening [4]. The limited lifespan of primary cells in in vitro culture is a significant factor that impacts the stability of the research findings. Telomerase is an enzyme involved in telomere synthesis, and is particularly important in synthesizing telomere repeat sequences and maintaining telomere length [8], whose introduction would help to promote cell immortalization. In this study, after introducing the exogenous hTERT gene by recombinant vector, BCF overcame the division limitation and was continuously passaged over 80 generations (Figure 2). Gene expression testing showed that the hTERT gene was introduced and stably expressed in the iBCF cell line, which promoted BCF cell immortalization (Figure 4). Actually, cell lines established using the telomerase introduction method maintained similar cell morphology to the primary cells, high proliferative activity, good wall apposition, and no tumorigenicity, and were considered close to the physiological pathway of species immortalization [31]. hTERT transfection has been the most commonly used method to construct immortalized cell lines in recent years [16,17].

Like most other cell lines, BCF is sensitive to nutrient concentrations, and it adapted to culture in the medium with 4.5 g/L glucose and 10% serum (Figure 3). Fibroblasts are characterized by the expression of some marker genes, such as VIM [26]. This study performed VIM expression detection and found that VIM is highly expressed in BCF, including pBCF and iBCF cells (Figure 5). In contrast, the epithelial cell marker CK18 showed low expression in both pBCF and iBCF, compared to in MAC-T (Figure 5), demonstrating that the immortalized BCF cell line constructed in this study belongs to fibroblasts. In addition, proliferation and viability detection showed that hTERT-induced iBCF exhibits faster growth rates and higher viability than primary BCF (Figure 6). Karyotype analysis showed that iBCF displayed the normal camel chromosome number (2n = 74) and structure as pBCF (Figure 7), consistent with previous reports [32]. Karyotyping is a process of preparing, arranging, and categorizing chromosomes using a cell culture technique in order to encounter various chromosomal abnormalities [33]. Together with the proliferation test, our study demonstrated that the introduction of telomerase promotes the immortalization of primary BCF, and enhances cell viability and proliferation activity; however, no evidence of any malignant phenotype was associated with the higher growth rate of iBCF.

In summary, an immortalized camel fibroblast cell line was successfully established using hTERT transfection in this study, which was named BCF23. The detailed properties of BCF23 are shown in Table 1. BCF23 was shown to have similar phenotypic and biological properties as primary BCF. This cell line showed a high proliferation rate and genetic stability without malignant phenotypes when cultured to higher generations in vitro. It helps to preserve the germplasm resources of Bactrian camel at the cellular level, and provides a cellular source for various functional studies of camels.

## Figures and Tables

**Table 1:** Comparison of characteristics of primary BCF and BCF23 cell lines.

Properties	pBCF	iBCF
Proliferative capacity	Weaker	Stronger
Passage number	About 10 passages	Infinite generations
Growth rate	Slow, 3–4 d	Fast, 2–3 d
Training difficulty	Difficult	Easy
The doubling time	Longer	Shorter
Optimal growth conditions (temp, CO_2_, media)	37 °C, 5% CO_2_ humidity incubator, 10% FBS, 4.5 g/L glucose DMEM	37 °C, 5% CO_2_ humidity incubator, 10% FBS, 4.5 g/L glucose DMEM
Biological characteristics	Approaching in vivo cell biology	Approaching in vivo cell biology
Genetic integrity	Unchanged	Unchanged
Karyotype	2n = 74	2n = 74
Malignant transformations or clonality	Negative	Negative

## Data Availability

The BCF23 cell line is available to all researchers. Please contact the corresponding author.
